# Identification of Two Long Noncoding RNAs, Kcnq1ot1 and Rmst, as Biomarkers in Chronic Liver Diseases in Mice

**DOI:** 10.3390/ijms25168927

**Published:** 2024-08-16

**Authors:** Shinya Yokoyama, Hisanori Muto, Takashi Honda, Yoichi Kurokawa, Hirotaka Ogawa, Riku Nakajima, Hiroki Kawashima, Hidenori Tani

**Affiliations:** 1 Department of Gastroenterology and Hepatology, Nagoya University Graduate School of Medicine, 65 Tsurumai-cho, Showa-ku, Nagoya 466-8560, Japan; yokoyama.shinya.w3@f.mail.nagoya-u.ac.jp (S.Y.); hisanori.muto@fujita-hu.ac.jp (H.M.); honda.takashi.y8@f.mail.nagoya-u.ac.jp (T.H.); h-kawa@med.nagoya-u.ac.jp (H.K.); 2 Department of Gastroenterology and Hepatology, Fujita Health University Bantane Hospital, 3-6-10, Otoubashi, Nakagawa-ku, Nagoya 454-8509, Japan; 3 Department of Bioscience and Biotechnology, Fukui Prefectural University, Eiheiji-cho, Fukui 910-1195, Japan; kurokawa@fpu.ac.jp; 4 Nagoya Industrial Science Research Institute, Nagoya 460-0008, Japan; ogawahirotaka09@gmail.com; 5 Department of Health Pharmacy, Yokohama University of Pharmacy, 601 Matano, Totsuka, Yokohama 245-0066, Japan; 201113_hj@yok.hamayaku.ac.jp

**Keywords:** mouse liver, hepatocellular carcinoma, lncRNAs, Kcnq1ot1, Rmst

## Abstract

This study investigates novel short-lived long noncoding RNAs (lncRNAs) in mice with altered expression in metabolic dysfunction-associated steatotic liver (MASH) and liver fibrosis. LncRNAs share similarities with mRNAs in their transcription by RNA polymerase II, possession of a 5′ cap structure, and presence of a polyA tail. We identified two lncRNAs, Kcnq1ot1 and Rmst, significantly decreased in both conditions. These lncRNAs showed dramatic expression changes in MASH livers induced by Western diets and CCl_4_, and in fibrotic livers induced by CCl_4_ alone. The decrease was more pronounced in liver fibrosis, suggesting their potential as biomarkers for disease progression. Our findings are consistent across different fibrosis models, indicating a crucial role for these lncRNAs in MASH and liver fibrosis in mice. With MASH becoming a global health issue and its progression to fibrosis associated with hepatocarcinogenesis and poor prognosis, understanding the underlying mechanisms is critical. This research contributes to elucidating lncRNA functions in murine liver diseases and provides a foundation for developing novel therapeutic strategies targeting lncRNAs in MASH and liver fibrosis, offering new avenues for potential therapeutic interventions.

## 1. Introduction

Metabolic dysfunction-associated steatotic liver disease (MASLD) has emerged as a prevalent cause of chronic liver ailments globally [1,2]. Some MASLD patients develop metabolic dysfunction-associated steatohepatitis (MASH), potentially leading to cirrhosis, hepatocellular carcinoma, and elevated liver-related mortality [3,4,5,6]. MASH is histologically characterized by steatosis, inflammation, hepatocyte injury (ballooning), and/or fibrosis [7]. The progression of fibrosis in MASH is particularly noteworthy due to its association with hepatocarcinogenesis and poor prognosis [8,9,10]. However, the underlying mechanisms of fibrosis progression remain poorly understood.

The liver plays a pivotal role in lipid homeostasis, orchestrating the synthesis, production, redistribution, and utilization of fatty acids as energy substrates. Disruption of any of these processes may lead to hepatic fat accumulation and MASLD development. Despite extensive research efforts, our comprehension of MASLD pathogenesis remains limited. There is an urgent need to identify efficacious therapeutic targets, particularly key pathogenic genes. A Western diet (WD) for the murine model, which is high-fat, high-fructose, and high-cholesterol, mimics fast food style diets that have been implicated in MASH pathogenesis in humans [11,12]. The WD induces obesity and insulin resistance in addition to MASH histology.

Carbon tetrachloride (CCl_4_) has long been utilized to induce liver injury and fibrosis in mice [13]. Previous research has suggested that multiple administrations of CCl_4_ combined with a high-fat diet induce oxidative stress, triggering inflammation and apoptosis, and ultimately leading to fibrosis development in mice [14]. Additionally, using a WD with a low, weekly dose of intraperitoneal CCl_4_ served as an accelerator [15].

Historically, messenger RNA (mRNA) has been considered the primary form of RNA, serving as a template for protein synthesis. Concurrent research has focused on fluctuations in protein levels. Also, the investigation of long noncoding RNAs (lncRNAs), which exceed 200 nucleotides in length initially lagged behind. However, recent comprehensive transcriptome analyses have unveiled the abundant transcription of lncRNAs within cells and their dynamic regulation of intracellular gene expression mechanisms [16]. These lncRNAs have been found to exert profound effects, reshaping our understanding of biological processes through their involvement in nuclear structures, chromatin remodeling, imprinting, transcription, translation, and epigenetic control [17,18]. lncRNAs share similarities with mRNAs in their transcription by RNA polymerase II, possession of a 5′ cap structure, and presence of a polyA tail. The key distinction between mRNAs and lncRNAs lies in the presence (mRNA) or absence (lncRNA) of a protein-coding region [19] Nevertheless, it is noteworthy that the functions of only a small fraction of non-coding RNAs have been elucidated thus far, with thousands potentially remaining functionally uncharacterized.

In 2012, two independent research groups, including the authors, conducted comprehensive measurements of cellular RNA half-lives, encompassing non-coding RNAs in mice and humans [20,21]. Their findings revealed that long-lived non-coding RNAs (half-life exceeding 4 h) predominantly comprise housekeeping-like RNAs, such as ribosomal RNAs, transfer RNAs, and nucleolar small RNAs. Conversely, short-lived non-coding RNAs (half-life less than 4 h) primarily consist of well-characterized regulatory long non-coding RNAs, including growth arrest specific 5 (GAS5) [22], HOX transcript antisense RNA (HOTAIR) [23], and nuclear paraspeckle assembly transcript 1 (NEAT1) [24]. These observations suggest a potential correlation between the temporal stability of lncRNAs and their functional significance. Consequently, previously unidentified transient lncRNAs may play modulatory roles in human cellular processes and serve as molecular indicators of cellular stress [25,26].

Next-generation sequencing data has highlighted the crucial role of lncRNAs across a spectrum of pathological conditions, encompassing infectious diseases, inflammatory disorders, and stress-related ailments. Dysregulation of lncRNAs is a common feature observed in various disease mechanisms. Elucidating the functions of lncRNAs could provide a molecular-level understanding of inflammatory processes, stress responses, and immune regulation, with particular emphasis on host-pathogen interactions. These insights may lead to the development of novel therapeutic approaches for combating infectious diseases. The hallmarks of infection typically include oxidative and endoplasmic reticulum stress, activation of the unfolded protein response, immune system activation, and inflammatory reactions [25,26].

Our investigation sought to identify novel short-lived lncRNAs in the livers of mice that exhibit altered expression in WD-induced metabolic dysfunction-associated steatotic liver with a control diet (CD). This focus stems from the increasing interest in elucidating the role of lncRNAs in mice and their potential as novel therapeutic targets. Our study identified two lncRNAs—Kcnq1ot1 and Rmst—in mice, that commonly exhibited dramatic expression changes in MASH livers induced by WD and CCl_4_ and in fibrotic livers induced by CCl_4_ alone.

## 2. Results

### 2.1. Evaluation of the Liver in Mice

**Figure 1:**
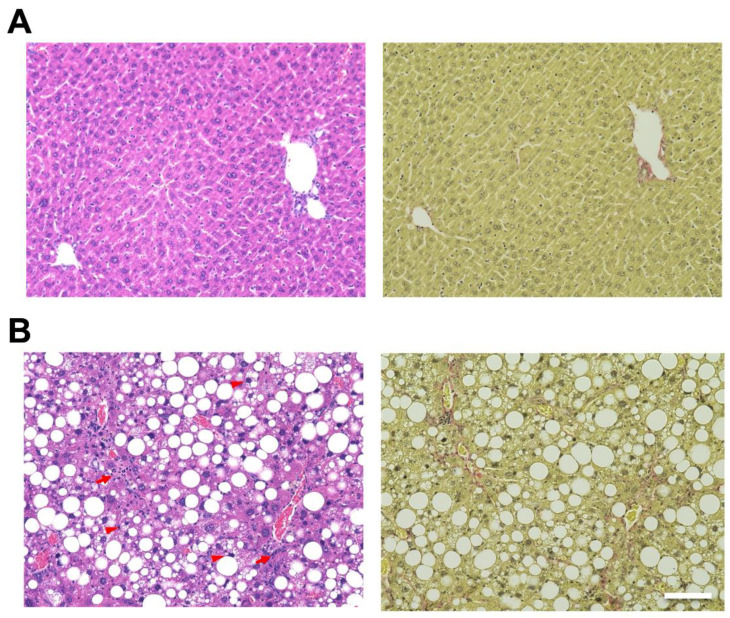
Mouse model fed Western diet (WD) and treated by CCl_4_-induced metabolic dysfunction-associated steatotic liver, which develops metabolic dysfunction-associated steatohepatitis (MASH). Control mice are fed in a control diet (CD). Hematoxylin and eosin (H&E) staining of livers from (**A**; **left**) CD-fed and (**B**; **left**) WD-fed mice. Sirius Red staining of livers from (**A**; **right**) CD-fed and (**B**; **right**) WD-fed mice. Scale bar: 100 μm. Arrowheads: hepatocyte ballooning, Arrows: infiltration of inflammatory cells.

**Figure 2:**
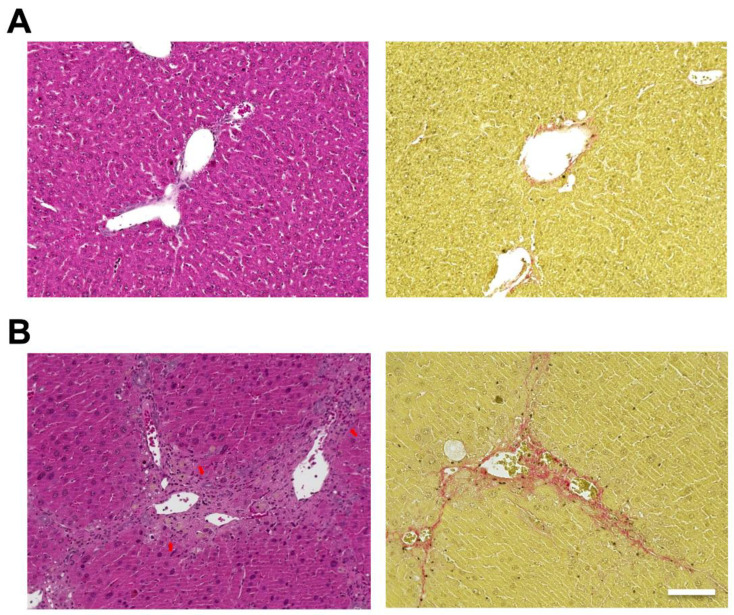
Carbon tetrachloride (CCl_4_) induced mouse model, which develops liver fibrosis. Control mice are fed in a control diet (CD) with no administration of CCl_4_. Hematoxylin and eosin (H&E) staining of livers from (**A**; **left**) control and (**B**; **left**) CCl_4_ injected mice. Sirius Red staining of livers from (**A**; **right**) control and (**B**; **right**) CCl_4_ injected mice. Scale bar: 100 μm. Arrows: infiltration of inflammatory cells.

Hematoxylin and eosin (H&E) staining of the liver showed hepatic steatosis and mild hepatocyte ballooning degeneration in WD-fed mice and a significant increase in Non-alcoholic Fatty Liver Disease Activity Score (NAS) [27] (Figure 1). Hepatic tissue from a mouse model of MASH showed about 60% steatosis, moderate inflammatory cell infiltration, and ballooning of hepatocytes (NAS: Steatosis: 2, Inflammation: 2, Ballooning: 1), which are similar to the characteristics of human MASH. Sirius red staining showed mild fibrosis around the portal area. The pathological findings in the liver fibrosis model mice using CCl_4_ with a control diet showed moderate fibrosis primarily around the portal areas, accompanied by infiltration of inflammatory cells (Figure 2). Fibrosis was more severe in the liver fibrosis model than in the MASH model.

### 2.2. LncRNA Expression Changes of the Liver in Mice

We chose ten lncRNAs that have brief half-lives (*t*_1/2_ ≤ 4 h) in mouse Neuro-2a cell line based on the following criteria [20]: (a) they exceed 200 nucleotides in length; (b) they are either highly or moderately expressed with a reads per kilobase of exon per million mapped reads greater than one according to RNA-Seq data in HeLa cells; (c) they can be found on the NCBI database; and (d) they do not intersect with known protein-coding gene regions (as shown in Table 1).

**Figure 3:**
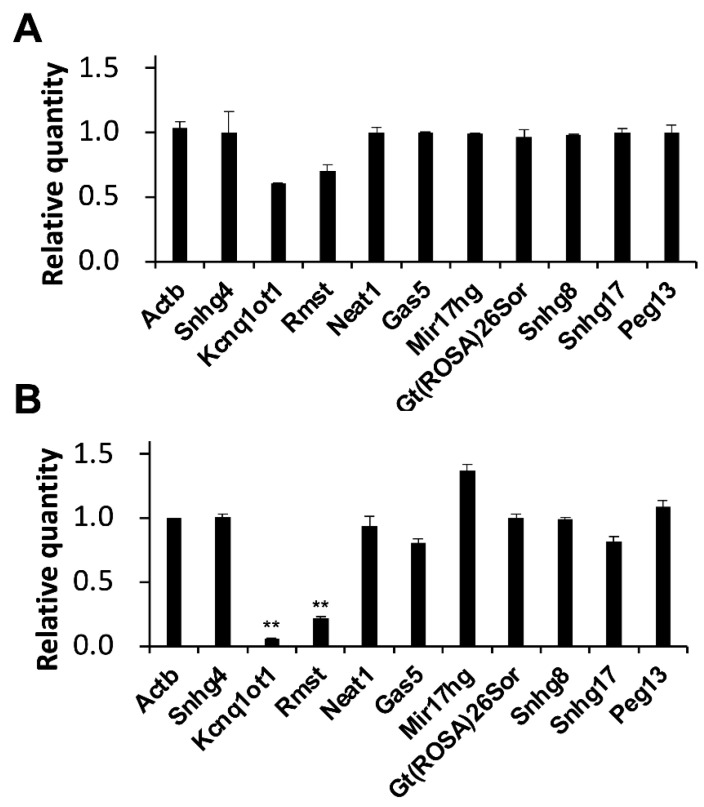
Alterations in lncRNA expression levels in model mice. Expression levels of the indicated RNAs were determined by RT-qPCR. GAPDH was used for normalization. Values represent the mean ± SD obtained from two (**A**) and four (**B**) independent experiments (** *p* < 0.01, Student’s *t*-test). The Y-axis indicated that the expression levels of WD-fed mice were divided by those of CD-fed mice. Thus, zero indicated that the RNA was not detectable and one indicated that there was no change in expression levels compared to the CD-fed mice.

Interestingly, decreases of 0.6- and 0.7-fold in the expression level of KCNQ1 overlapping transcript 1 (Kcnq1ot1), and rhabdomyosarcoma 2 associated transcript (Rmst) were observed in MASH model mice (Figure 3A). Moreover, significant decreases of 0.1- and 0.2-fold in the expression level of Kcnq1ot1 and Rmst were observed in liver fibrosis model mice (Figure 3B).

The basic information of the two lncRNAs is as follows. Kcnq1ot1, genome location: chr7; 142,766,848–142,850,284; length: 90,612 nt; NCBI RefSeq ID: NR_001461; representative previous research: [28]. Rmst, genome location: chr10; 91,917,608–92,001,040; length: 2687 nt; NCBI RefSeq ID: NR_028262; representative previous research: [29].

## 3. Discussion

Our research revealed significant reductions in two short-lived lncRNAs, Kcnq1ot1 and Rmst, in chronic liver diseases, encompassing both hepatic steatosis and fibrosis. These findings suggest a potentially crucial function of these lncRNAs in murine chronic liver diseases. Notably, the lncRNAs decrease was more pronounced in hepatic fibrosis mice than MASH mice, which may reflect the severity of liver fibrosis. These lncRNAs show as decreased in different types of mouse models, reflecting the severity of liver fibrosis, and indicating their potential as liver fibrosis biomarkers which is an important prognostic factor in MASH and other liver diseases.

Recent investigations have shown KCNQ1OT1/Kcnq1ot1 to be upregulated in various malignancies, including breast and liver cancers, promoting disease progression and metastasis [30,31]. KCNQ1OT1/Kcnq1ot1, an lncRNA transcribed from the opposite strand of KCNQ1 within the CDKN1C/KCNQ1OT1 cluster, has been implicated as a critical factor in cancer development and progression. This lncRNA interacts with microRNAs, such as miR-506, miR-204-5p, or miR-107 in cancer cells, to modulate cellular processes and contributes to cancer development [32]. While previous studies consistently reported KCNQ1OT1/Kcnq1ot1 overexpression in diverse cancers, our results demonstrate decreases in its expression [30,31,32,33,34]. These contrasting findings highlight KCNQ1OT1/Kcnq1ot1 as a potential target for cancer therapeutics.

Conversely, RMST/Rmst expression is elevated in gastric cancer tissues [35], but diminished in thyroid [36] and colorectal cancers [37]. RMST/Rmst exhibits tissue-specific expression, notably in brain tissues and vascular endothelial cells, and plays a significant role in neuronal differentiation through interaction with SRY-box 2 (SOX2). In cancer, RMST predominantly functions as a tumor suppressor, with context-dependent roles observed across different cancer types. RMST is also implicated in neurological disorders, cardiovascular diseases, and Hirschsprung’s disease. Mechanistically, RMST acts as a competing endogenous RNA and a transcriptional regulator, interacting with various microRNAs and proteins to modulate gene expression. Research has shown that RMST/Rmst can interact with microRNAs, such as miR-1251, miR-145, or miR-1202 in cancer cells, modulating their activity and impeding tumor progression by suppressing cancer cell proliferation and metastasis [35,36,37,38,39]. These observations suggest that RMST/Rmst may also be a promising candidate for cancer therapy development.

This study is limited by the lack of elucidation of the mechanisms by which Kcqn1ot1 and Rmst respond to liver disease. Future research should focus on unraveling this mystery through detailed analysis of these two lncRNAs. We anticipate that this study will contribute to understanding the role of lncRNAs in murine diseases. Our findings may provide a foundation for developing novel therapeutic strategies against MASH and other forms of liver fibrosis, as lncRNAs represent attractive interventional targets. Potential approaches include the use of antisense oligonucleotides, small interfering RNAs, and CRISPR-Cas9 gene editing techniques.

## 4. Conclusions

In this study, we identified two novel short-lived lncRNAs, Kcnq1ot1 and Rmst, which show significant expression changes in mouse models of chronic liver diseases, including MASH and fibrosis. These lncRNAs exhibited more pronounced decreases in hepatic fibrosis compared to MASH, suggesting their potential as biomarkers for liver fibrosis severity. Our findings contrast with previous studies on KCNQ1OT1/Kcnq1ot1 in various cancers, highlighting its complex role in different pathological contexts. Similarly, RMST/Rmst shows varied expression patterns across different cancer types, indicating its potential as a therapeutic target. While our study is limited by the lack of mechanistic insights, it provides a foundation for future research into the role of lncRNAs in liver diseases and their potential as therapeutic targets. Further investigation using techniques such as antisense oligonucleotides, siRNAs, and CRISPR-Cas9 may lead to novel treatment strategies for MASH and liver fibrosis.

## 5. Materials and Methods

### 5.1. Murine Model

Nine-week-old male C57BL/6 J mice from Japan SLC (Fukuoka, Japan) were used for these experiments. Mice for the MASH model were fed a WD containing 21.1% fat, 41% sucrose, and 1.25% cholesterol by weight (Teklad Diets; Envigo, Indianapolis, IN, USA), as well as a high sugar solution (23.1 g/L d-fructose (Sigma-Aldrich, Burlington, MA, USA) and 18.9 g/L d-glucose (Sigma-Aldrich)). CCl_4_ (Wako, Tokyo, Japan) at a dose of 0.2 μL (0.32 μg)/g of body weight was injected intra-peritoneally once per week over the course of 12 weeks. Liver fibrosis model mice were fed a control diet and injected with 1.2 μL (1.9 μg)/g of CCl_4_ (Wako) subcutaneously into the dorsal region twice a week over the course of 12 weeks. The experimental protocol involving animals was approved by the Institutional Animal Care and Use Committee of Nagoya University on 6 March 2023 (M220028-002). Furthermore, all experiments performed in this study were performed by the principles of care and use of experimental animals as prescribed by the National Institute of Health and conforming to the ARRIVE guidelines.

### 5.2. Histological Analyses

Formalin-fixed, paraffin-embedded liver samples were cut into 4-μm-thick sections and stained with hematoxylin–eosin (H&E) and Sirius red. NAFLD activity score (NAS) was calculated as the sum of individual scores for steatosis (0–3), ballooning (0–2), and lobular inflammation (0–3). Sirius red positive area and lipid droplet area were calculated at 20× magnification using a BZ-X800 microscope (Keyence, Osaka, Japan).

### 5.3. RNA Isolation

Total RNA was extracted from cells with TRIzol Reagent (Thermo Fisher Scientific, Waltham, MA, USA), in accordance with the manufacturer’s instructions. Add 1 mL of TRIzol Reagent (Thermo Fisher Scientific) per 50–100 mg of tissue to the sample and homogenize using a homogenizer. Incubate for 5 min to allow complete dissociation of the nucleoproteins complex. Add 0.2 mL of chloroform per 1 mL of TRIzol Reagent (Thermo Fisher Scientific) used for lysis, securely cap the tube, then thoroughly mix by shaking. Incubate for 2–3 min. Centrifuge the sample for 15 min at 12,000× *g* at 4 °C. The mixture separates into a lower phenol-chloroform, an interphase, and a colorless upper aqueous phase. Transfer the aqueous phase containing the RNA to a new tube by angling the tube at 45 °C and pipetting the solution out. Add 0.5 mL of isopropanol to the aqueous phase, per 1 mL of TRIzol Reagent (Thermo Fisher Scientific) used for lysis. Incubate for 10 min at 4 °C. Centrifuge for 10 min at 12,000× *g* at 4 °C. Total RNA precipitate forms a white gel-like pellet at the bottom of the tube. Discard the supernatant with a micropipettor. Resuspend the pellet in 1 mL of 75% ethanol per 1 mL of TRIzol Reagent (Thermo Fisher Scientific) used for lysis. Vortex the sample briefly, then centrifuge for 5 min at 7500× *g* at 4 °C. Discard the supernatant with a micropipettor. Air dry the RNA pellet for 5–10 min. Resuspend the pellet in 20 µL of RNase-free water by pipetting up and down. The isolated RNA was reverse transcribed into cDNA using PrimeScript RT Master Mix (Perfect Real Time) (TaKaRa, Tokyo, Japan).

### 5.4. Quantitative PCR (RT-qPCR)

The cDNA was amplified using the primer sets listed in Table 2, and the levels were normalized relative to glyceraldehyde-3-phosphate dehydrogenase (Gapdh) mRNA. Relative RNA quantities were calculated as mouse model values normalized relative to CD-fed mouse values. We have normalized the changes in Actb based on Gapdh, that is, normalized so that the relative quantity becomes 1. The sample numbers were three in each gene (N = 3). THUNDERBIRD SYBR qPCR mix (Toyobo, Osaka, Japan) was used per the manufacturer’s instructions. For significance testing, Student’s *t*-test was used. RT-qPCR analysis was performed using a Quantstudio 7 Flex (Thermo Fisher Scientific). For RT-qPCR, the sample volume was 10 μL, and three independent replicates were performed. Additionally, there were no apparent error values, and all obtained data were used.

## Figures and Tables

**Table 1:** The 10 short-lived lncRNAs that were investigated in this study.

Name	Accession No.	Length (nt)	*t*_1/2_ *
Snhg4	NR_038073	1050	0.32
Kcnq1ot1	NR_001461	90,612	1.60
Rmst	NR_028262	2687	1.38
Neat1	NR_003513	3190	0.01
Gas5	NR_153812	501	0.18
Mir17hg	NR_029382	2339	0.07
Gt(ROSA)26Sor	NR_027010	613	0.12
Snhg8	NR_028574	785	0.14
Snhg17	NR_015463	3175	0.48
Peg13	NR_002864	4723	0.67

* These values are taken from a previous report [20].

**Table 2:** Primer pairs for RT-qPCR.

Name	Sence Sequence (5′-3′)	Antisence Sequence (5′-3′)
Gapdh	TGGTGAAGCAGGCATCTGAG	TGAAGTCGCAGGAGACAACC
Actb	CCAGCCATGTACGTAGCCAT	CGGAGTCCATCACAATGCCT
Snhg4	GCATTTTCAGAGTCAACATTCCCA	GAAGCCTCAAGACCTAAACTCCA
Kcnq1ot1	GGCGCAGGTCTGTAATCGTA	CCCAGCAAGGTCCTGAACAT
Rmst	GCAGCTAAAGGACAGGAGCA	TTAACTCGCAGTTGGTGGCA
Neat1	AGTGTGAGTCGTAGCAGTGC	TCACTGTGTAGGCGTCAACC
Gas5	ATGGTGGAGTTTGAGGCTGG	CAAGCAAGCCAGCCAAATGA
Mir17hg	GCACTTGTTCAGTTCCGCAC	CCACAGCTGTTTTGCCAACA
Gt(ROSA)26Sor	GCCACACATGTCCCATTCCA	AACTTTCCAACCCACCACCC
Snhg8	CACAAGGTGGCTATGGTGCT	TCGTCGCGCTAACCTTCAC
Snhg17	TGTCTCCCAAGAGCTCCAGT	ATCCTGCTATGGCCTCCTGA
Peg13	GATTCCACTTGGGTGCCTCA	GACGTCTGCCTGGTCTCTTC

## Data Availability

The raw data supporting the conclusions of this article will be made available by the authors on request.
